# Effect of Purple Eggplant Flour on Physicochemical, Lipid Oxidation, and Sensory Properties of Low-Fat Beef Patties

**DOI:** 10.1155/2022/9753201

**Published:** 2022-02-02

**Authors:** Thanaporn Bunmee, Phatthawin Setthaya, Niraporn Chaiwang, Thanikarn Sansawat

**Affiliations:** ^1^Division of Animal Science, School of Agriculture and Natural Resources, University of Phayao, 56000, Thailand; ^2^Science and Technology Research Institute, Chiang Mai University, 50200, Thailand; ^3^Department of Agricultural Technology and Development, Faculty of Agricultural Technology, Chiang Mai Rajabhat University, 50300, Thailand; ^4^Division of Food Science and Technology, School of Agriculture and Natural Resources, University of Phayao, 56000, Thailand

## Abstract

This study examines the implications of PEF as an alternative fat replacer on nutritional composition, display storage stability, product quality, and its practical application for beef patties. Four different beef patties were formulated with 0, 2.5, 5.0, and 7.5% PEF. Addition of the PEF in beef patties resulted in a significant increase in moisture, ash, and total dietary fiber while decreasing protein and fat contents. The cooking yield, moisture, and fat retention of the PEF beef patties were significantly higher than the control patty. The tenderness and juiciness scores of the PEF beef patties were significantly increased compared to the control. The lightness and redness values of raw patties were superior to the control during storage time. The amounts of thiobarbituric acid-reactive substances (TBARS) were lower in PEF beef patties than the control patties during 7 days of storage at 4°C. These results suggested that PEF could be used as a natural antioxidant fat replacer in beef patties without losing sensory and visual quality. In addition, the utilization of PEF may improve nutritional values including dietary fiber and display storage stability in beef patties.

## 1. Introduction

Consumers are becoming more discretionary and complicated in their decisions, influenced by economic condition, health concerns, and change in the meat industry regarding nutritional composition. High fat content has been involved in many health issues including diabetes, respiratory disease, obesity, hypertension, cardiovascular disease, and various cancers. As consumers become more health conscious, it will be ideal to reduce fat content, enhance dietary fiber, and increase palatability in meat products including meat patties. Unfortunately, reducing fat content can cause quality, visual juiciness/tenderness, and yield problems [1]. It has been known that fat in meat products improves water holding capacity, texture, sensory attributes, cooking yields, emulsion stability, and consumer acceptance [2]. Thus, fat replacers should be recognized as a sensorial and functional enhancer with healthy and safety benefits. Functional components of fat replacers can have a significant role in the promotion of well-being and preventing disease. To meet the similar properties of fat, scientists evaluated series of ingredients as potential functional ingredients such as synthetic compound, protein-based substance including collagen, soy protein, egg white, whey protein [3], fat-based substance including soy lecithin [1], and carbohydrate-based substance including starch, pectin, cellulose, and modified starch [4]. Moreover, various types of starch and dietary fiber from cereal and legumes have been utilized in an attempt to improve nutritional quality along with cost reduction of meat-based patties [5].

Eggplant (*Solanum melongena* L.), known as aubergine in Europe, is one of the most important vegetables commonly grown in tropical and subtropical regions. Eggplant is an excellent source of mineral, dietary fiber, and phytochemical that contribute to human health improvement. The color of eggplant usually varies from creamy to greenish or purplish caused by the level of anthocyanin. Purple eggplant is classified as one of the healthiest foods due to low calorie and high phenolic content as a powerful free-radical scavenger [6]. Chlorogenic acid, a major phenolic compound, has anti-inflammatory property and cardioprotective function [7]. Purple eggplants also have bactericidal activity against *E. coli*, *S. aureus*, *B. subtilis*, and *Pseudomonas* sp. [8]. Nasunin and nasurin, phenolic compounds in eggplant skin, are key phytochemicals considered as nutraceuticals. Nasunin has a high superoxide radical scavenging activity helping antiperoxidation and antiaccumulation of reactive oxygen species in cell [9]. Consequently, purple eggplant can serve as a natural antioxidant in meat patties. Similarly, antioxidant capacity is one of the common functions in functional food [10]. Functional food is defined as a conventional food that consists of bioactive substance or/and physiological compound that provides health benefit beyond basic nutrient. Hence, the nutritional value of patty can be enhanced by supplementing with other nutrient source substitutes, especially purple eggplant. Purple eggplant can be processed into flour and incorporated into beef patties.

Purple eggplant flour (PEF) may provide health benefits by lowering calorie and fat contents, increasing dietary fiber, and providing antioxidant in beef patties. Moreover, PEF is considered to be another source of income for farmers while it is also a cheap, readily available, and natural antioxidant supplement for the meat. However, there is no research that has been conducted for the effect of PEF on the quality of beef patties. We hypothesized that PEF can be used as a functional ingredient to improve the quality and nutritive properties of beef patties without impairing flavor. Therefore, the aim of this study was to evaluate the effects of fat replacement by PEF on nutritional, physicochemical, lipid oxidation, and sensory characteristics of low-fat beef patties.

## 2. Materials and Methods

### 2.1. Preparation of Purple Eggplant Flour

Purple eggplant with uniform size, ripeness, and freshness was obtained from a local market in Phayao province, Thailand. Purple eggplant flour (PEF) was prepared according to the protocol of Uthumporn et al. [11], with a slight modification. Purple eggplant was washed thoroughly under running tap water to remove dirt and soil. The eggplant with peel was sliced and dried in a hot air oven (INS-0204, Memmert, Germany) for 72 hours at 45-50°C which can retain the content of phenolic compounds [6]. The dried purple eggplant was then finely ground and sieved to make to PEF using a heavy-duty mixer grinder (HC-350, Zhejiang, China) and a 250 *μ*m sieve on a vibrator shaker (CY-1800, Henan, China). PEF was vacuum-sealed in a polypropylene plastic bag, kept in a dark container, and stored at -20°C prior to use.

### 2.2. Preparation of Raw and Cooked Beef Patties

Fresh boneless chuck (m*. supraspinatus*) and beef fat were purchased from a local supermarket in Phayao province, Thailand. The beef chuck was trimmed of connective tissue and visible fat before mincing with sodium chloride and icy water with a grinder through a 6 mm plate (AW114, Valencia, Spain). Beef fat (13.2% moisture, 85.65% fat) was ground through a 4 mm plate. Four groups of patties (about 3 kg per group) were prepared with 0 (control), 2.5, 5, and 7.5% PEF by weight using the formulations in Table 1. The PEF weight differences were adjusted with beef fat. Other required ingredients were added to each batch and mixed manually for 10 min. The mixture was formed into a patty (8.5 cm internal diameter, 80 g weight, and 1.5 cm thickness) by using a patty. Finally, the patties were placed on polystyrene trays and wrapped individually with cling film. Raw patty samples were taken for color and oxidative stability trial on the same day. The rest of patty samples were vacuum-packed and kept at -20°C for further analysis.

In addition, beef patties were cooked, after spraying vegetable oil, in a Teflon-coated pan until the internal temperature reached 75 ± 2°C. The internal temperature was measured at the center of the patty by inserting a thermocouples probe (Consort T851, Cohasset, MA, USA). The cooked patties were packed in food grade polyethylene bags and allowed to cool to 25°C before testing. The cooked patties were subsequently evaluated for their different nutritional compositions, cooking properties, instrumental textural properties, and sensory characteristics.

### 2.3. Retail Display Studies

Raw beef patties were aerobic-packed in polystyrene trays and stored for 7 days on shelves (4°C) in a retail refrigerated display case (SBL-1500 Shabu, Sanden Intercool, Thailand) under 1,614 lx of continuous daylight (Phillips, Thailand). On the days of 0, 1, 3, 5, and 7 in the display case, samples of each treatment were taken for the analyses of storage loss, color stability, and lipid oxidation.

### 2.4. Proximate Analysis

Proximate analysis of PEF, raw, and cooked beef patties were determined for moisture, protein, fat, ash, and crude fiber contents using the method of the Association of Official Analytical Chemists [12]. The carbohydrate content was determined through the following equation:

(1) $$\begin{array}{c} \%\text{Carbohydrate}\left(\text{DM}\right)=100\%-\left(\%\text{moisture}+\%\text{ash}+\%\text{fat}+\%\text{protein}+\%\text{crude fiber}\right). \end{array}$$

Total energy values (kcal) were calculated on the basis of 100 g portion using Atwater value for protein (4.02 kcal/g), fat (9 kcal/g), and carbohydrate (3.87 kcal/g) as described by Mansour and Khalil [13]. The calorie values were estimated values. All analysis was performed in triplicate.

### 2.5. Measurement of Cooking Properties

The cooking yield of five patties/treatment was measured by the patty weight difference (%) before/after cooking and cooled to 25°C. Cooking yield, moisture, and fat retention were calculated according to the method of Sánchez-Zapata et al. [14] as follows:

(2) $$\begin{array}{c} \text{Cooking yield}\left(\%\right)=\left[\frac{\left(\text{cooked patty weight}\right)}{\left(\text{raw patty weight}\right)}\right]\times 100,\\ \text{Moisture retention}\left(\%\right)=\frac{\left(\%\text{yield}\times \%\text{moisture in cooked patty}\right)}{100},\\ \text{Fat retention}\left(\%\right)=\left[\left(\text{cooked patty weight}\right)\times \frac{\left(\%\text{fat in cooked patty}\right)}{\left(\text{raw patty weight}\right)}\times \left(\%\text{fat in the raw patty}\right)\right]\times 100. \end{array}$$

The diameter and thickness of beef patties were measured with a digital caliper before and after cooking. All determinations were performed in three replications for each treatment. The following calculations were performed to estimate the change in the diameter and thickness of beef patties:

(3) $$\begin{array}{c} \text{Diameter reduction}\left(\%\right)=\left[\frac{\left(\text{raw patty diameter}–\text{cooked patty diameter}\right)}{\text{raw patty diameter}}\right]\times 100,\\ \text{Thickness reduction}\left(\%\right)=\left[\frac{\left(\text{raw patty thickness}–\text{cooked patty thickness}\right)}{\text{raw patty thickness}}\right]\times 100. \end{array}$$

### 2.6. Instrumental Textural Property Analysis

Texture profile analysis and Warner-Bratzler shear force of raw and cooked samples were performed at room temperature using a TA-XT texture analyzer (Stable Micro Systems, Godalming, UK). Samples were cooked according to the procedures previously described. Raw and cooked beef patties (5.08 cm diameter and 1.5 cm thickness) were compressed twice to 50% of their original height with a 9 cm flat end steel plunger at a speed of 100 mm/min in a one-cycle compression test. A force-time graph was generated, and textural properties were evaluated for hardness (raw beef patties), springiness, chewiness, cohesiveness, and firmness of cooked beef patties obtained. The shear force of the cooked beef patties was determined using a Warner-Bratzler blade attached to the texture analyzer, operated at 3 mm/min and 5 g of trigger force [15]. The crosshead speed of 25 cm/min and a 50 kg load cell were used. Three replicate samples (2.5 cm wide × 2 cm height × 5 cm long) were taken for each treatment. The shear force value was recorded at the maximum peak force of the graph and expressed as Newton (N).

### 2.7. Color Measurement

Color evaluation (CIE L∗, a∗, and b∗) was performed on the surface of raw beef patties using a Minolta Chroma Meter (CR 400, Minolta, Osaka, Japan; parameter settings: diffuse illumination, 10° viewing angle, measuring area, ø 11 mm). Samples were taken for color measurement at days 0, 1, 3, 5, and 7 of retail display at 4°C. Before measuring, samples were exposed to oxygen for 1 h at 25°C to allow blooming. Six readings per sample were taken, and the mean value was calculated for each of the three replications.

### 2.8. Lipid Oxidation Measurement of the Beef Patties during Retail Display Storage

Lipid oxidation of the beef patties was assessed by measuring thiobarbituric acid reactive substances (TBARS) using the method described by Reitznerová et al. [16] with slight modification. The TBARS value was expressed as *μ*g of malondialdehyde (MDA)/g sample. Briefly, a meat sample (2 g) was homogenized with 20 mL trichloroacetic acid solution (20% *w*/*v*) and 5 mL of butylhydroxytoluene (0.8% *v*/*v*) in hexane for 2 min using a homogenizer (Nissei AM-8 homogenizer; Nissei Corporation, Tokyo, Japan) and then centrifuged at 1,600 × g for 10 min (MX-305; Tomy Seiko, Tokyo, Japan). The supernatant (2 mL) was mixed with 1.5 mL ice-cold thiobarbituric acid solution (0.1% *w*/*v* in double-distilled water), and the lower layer was filtered through Whatman No.1 filter paper. For derivatization, aliquots of the filtrate (500 *μ*L) were transferred into a vial and added 50 *μ*L DNPH reagent (31 g of dinitrophenylhydrazine dissolved in 10 mL of 2.0 M HCl and incubated for 30 min at room temperature in the dark). HPLC (Thermo Fisher Scientific, Waltham, MA, USA) was used to separate and quantitate the contents of the MDA–DNPH complex. Chromatographic separation was achieved on a Polaris C18-A chromatographic column (particle size, 5 *μ*m; column size 250 mm × 4.6 mm; Varian, Santa Clara, CA, USA). Samples were isocratically eluted with a mixture of 0.2% (*v*/*v*) glacial acetic acid in deionized water and acetonitrile (61 : 39, *v*/*v*) at a flow rate of 1 mL/min at 25°C. The injection volume was 20 *μ*L, and the DAD detector was set at 307 nm. Analyses were performed using a Chromeleon Chromatography Data System, Version 7.2 (Thermo Fisher Scientific, Waltham, MA, USA) for collecting and processing data. The peak area of the MDA–DNPH curve was plotted against the concentration to obtain the calibration graph. The MDA–DNPH peak was identified by the elution profile of the authentic standard peak identification in meat samples that were performed by comparison of the retention time with the standard. All samples were determined MDA by HPLC (Thermo Fisher Scientific, Waltham, MA, USA) at 0, 1, 3, 5, and 7 days under cool retail display.

### 2.9. Trained Sensory Evaluation

Cooked samples with randomly coded numbers were served warm to the panelists. Ten trained panelists rated 20 samples per session according to the standard outlined by the AMSA [17]. The 9-point scale was used for sensory evaluation. The patties were evaluated for subjective measures in terms of hardness (1 = extremely hard, 9 = extremely soft), juiciness (1 = extremely dry, 9 = extremely juicy), off-flavor (1 = no off-flavor, 9 = extremely high off-flavor), and degree of satisfaction for its appearance, color, odor, texture, taste, and overall acceptability (1 = extremely dislike, 9 = extremely like). The panelists were seated in individual booths under normal fluorescent lighting. Water and bread were served between the samples to clean the mouths of the panelists.

### 2.10. Statistical Analysis

Results are presented as mean ± standard deviation. Statistical analysis of data was carried out using the SAS software package (SAS Institute, Cary, NC, USA). The obtained data were analyzed using a complete randomized design. Analysis of variance (ANOVA) was used to determine the significant difference between the results. Duncan's multiple range test was used to separate the mean with a significant level of 0.05.

## 3. Results and Discussion

### 3.1. Nutritional Compositions of PEF, Raw, and Cooked Beef Patties

The nutritional composition of PEF was carried out on wet basis and is reported in Table 2. The moisture content of PEF was 5.21%, lower than that reported by Uthumporn et al. [6], probably due to the lower temperature used in drying process. The flour having a moisture content between 9 and 10% showed a better storage stability than flour with higher moisture content [18]. Apparently, PEF is suitable for use and appropriate for long-lasting storage. The protein content of PEF was lower than that reported by Uthumporn et al. [6]. This might be due to the different fertilizer used in eggplant cultivation. The addition of animal manure to soil as fertilizer can increase the protein content of plant [19]. The fat content of PEF was 1.87%, similar to the results reported by Uthumporn et al. [6]. PEF had lower fat content compared to rice flour, wheat flour, and soybean flour [20] while the ash and crude fiber content were 7.45% and 12.64%, respectively. This data showed that the PEF is the good source of mineral and dietary fiber, with low-fat level. The carbohydrate content and total energy value of PEF were 62.35% and 322.72 kcal, similar to the carbohydrate content obtained by Hussain et al. [21]. The total energy value of flour depends on the amounts of carbohydrate and fat in flour.

The nutritional compositions of raw beef patties were affected by PEF levels (Table 2). The protein content of PEF beef patties was lower (*p* < 0.05) than the control patties, with no difference observed for moisture (*p* > 0.05). This might be due to the dilution effects from the larger amount of fiber and carbohydrate content of PEF used in patties. Similar results of chicken nuggets that added oat fiber [22], pork burger that added albedo-fiber powder [23], and beef burger that added pineapple byproduct were reported by Selani et al. [24]. The fat content of raw patties decreased (*p* < 0.05) with the increase of PEF while ash and crude fiber contents increased, potentially due to the high mineral and dietary fiber in PEF. A similar trend was observed after cooking, showing the reduction of protein and fat contents with no difference in moisture content (*p* > 0.05) among the treatments (Table 2). Cooked beef patties had a higher energy value than raw beef one due to the reduction in moisture content during cooking. The energy value of the control beef patties was the highest, regardless of cooking. The energy values were largely affected by fat that yields 9 kcal or 2.5 times more than carbohydrate and protein. The substitution of fat by PEF reduced the energy value of patties compared to the control patties due to the low energy of PEF. These results agree with the findings that the calorie values of frankfurters with rice bran fiber were significantly lower than those of control ones due to reduced fat [25, 26]. According to the Thai Dietary Reference Intake (Thai DRIs), the recommended dietary energy for 19 to 24 years old men and women is 1800 and 1500 kcal, respectively. The results from this study indicated that consuming 100 g of PEF beef patties (7.5%) would provide 12.83 and 15.41% of the dietary energy requirement of adult men and women, respectively.

### 3.2. Cooking Properties of Cooked Beef Patties

The addition of PEF significantly (*p* < 0.05) affected the cooking properties of the beef patties (Table 3). Increasing PEF level incorporation resulted in improving cooking yields (*p* < 0.05) of the beef patties, probably due to their water and fat binding properties. The highest fat loss was found in the patty with the highest fat content, which could be due to high amount of fat melting. In addition, the differences in cooking yield between treatments might be caused by water evaporation and fat outflow from beef patties during cooking [14]. Moisture and fat retention of beef patties are important because it affects desirable texture, juiciness, flavor, and palatability of cooked product. The lowest moisture was observed in the control that increased proportionally with the addition of PEF (*p* < 0.05). Meanwhile, water absorption capacity of beef patties increases with the increase of dietary fiber that contains a lot of hydroxyl (OH) group for hydrogen bonds with water molecules [11]. These results are in agreement with Essa and Elsebaie [27] who reported that there was an increment in the water retention values when the levels of date pit powder were increased in beef burger. The moisture retention of beef burger containing pea starch and pea fiber was significantly improved compared to control wheat crumb burger [28]. Water losing of beef burger was about 80% from during pan-frying because of the breakdown of myofibrils and connective tissue [29].

Maintaining fat within the meat product during cooking is necessary to guarantee organoleptic quality and acceptability [30]. The fat retention of the beef patties with PEF was significantly higher than the control patties (*p* < 0.05). In addition, the fat retention of the beef patties increased as the PEF addition increased (*p* < 0.05). This most likely occurred because PEF contains dietary fiber which has high binding capacity to fat. Similar results were reported by Kumar and Sharma [31] who also observed a significant increase of fat after cooking ground pork that was made with carrageenan, presumably due to the fat binding ability of carrageenan. According to Tarté [32], the addition of nonmeat ingredients such as cereal, plant fiber, and starch in a burger formula is used extensively as a binder, a filler, and an extender to create meat matrixes with improving water and fat holding capabilities. In agreement with Anderson and Berry [33] who reported that high fiber-base ingredient such as cellulose and inner pea fiber had significantly higher fat holding capacity than soy protein concentrate or gum, they documented that the mechanism of improving fat retention was predominantly driven by the ability of matrix formation with protein after swelling of the fiber and starch structure. Moreover, melting fat globules during cooking from beef patties with high fat content induced the leakage of fat from inside to outside of the beef patties [34]. This resulted in low-fat retention of the patties. Thus, the addition of PEF to beef patties can reduce the fat leakage and improve cooking yield. The diameter of cooked PEF beef patties was significantly (*p* < 0.05) larger than the control. This could be due to the binding property of PEF, which held the particles together and resisted to the changes in dimension of the product. Denaturation of meat protein during cooking causes patty shrinkage and loss of water and fat [28]. In agreement with this study, the diameter reduction of beef burger having pea and wheat fiber was lower than the control [35]. It was also found that the difference in reduction of diameter and thickness could be related to water and fat absorption properties of nonmeat ingredients [4, 14].

### 3.3. Instrumental Texture Properties

Food texture plays an important role in influencing the consumer satisfaction and the decision to repurchase the products [36]. Textural properties of raw and cooked PEF beef patties are shown in Table 4. The addition of PEF to beef patties significantly (*p* < 0.05) increased the hardness of raw patties and chewiness and cohesiveness of cooked patties. These results are probably due to the lower fat with higher PEF. Similarly, the addition of pea fiber increased the firmness value of beef burger [28]. The significant increase in hardness with dietary fiber was due to its improved binding ability and water holding capacity [23]. On the other hand, dietary fiber could have interfered the formation and stability of meat matrix and led to lower hardness [37]. Some reports also indicated that the textural properties such as hardness were not affected by the addition of plant-base fiber [14]. Increasing PEF levels was associated with increased chewiness and cohesiveness that was supported by Salcedo-Sandoval et al. [38]. These findings are consistent with the results of the patty with high water, low meat, and high dietary fiber contents in the formulation. Different results have been documented for the effect of dietary fiber addition on textural properties of meat product depending on the type and amount of fiber used. For the Warner-Bratzler shear force value which was widely used for meat tenderness assessment [39], there were no differences among all formulas. Unlike our results, beef burger with oatmeal had lower shear force value compared to the control [40]. The reason for the inconsistent results is not clear, but it may be due to multiple factors that affect the hardness of meat products such as water content, fat content, raw meat quality, and cooking methods.

### 3.4. Color Stability and Lipid Oxidation of the Beef Patties during Retail Display Storage

The color of the meat product is one of the most significant factors affecting consumer buying decisions. Meanwhile, the addition of nonmeat ingredients to meat products could lead to desirable and undesirable changes in color [34]. The L∗ (lightness) and a∗ (redness) value decreased (*p* < 0.05) as PEF level increased (Table 5). This lightness reduction might be due to the unique dark purple color of anthocyanins in purple eggplant skin. In addition, protein oxidation in emulsified cooked patties with fruit extracts influences the color deterioration during chilled storage [41]. A similar finding of lower L∗ was reported in PEF cookies [11]. In addition, the significant differences (*p* < 0.05) were found in L∗ and a∗ values due to the storage condition of retail display. The L∗ value of all beef patties increased progressively with increasing storage time. Regardless of storage day, the control patties had the highest a∗ value, whereas the 7.5% PEF patties had the lowest a∗ value (*p* < 0.05) that gradually decreased as the storage was extended. An increased PEF level led to an increase in the b∗ value in the patties. On the initial day, the control beef patties had lower L∗ and higher a∗ than those of the 2.5, 5, and 7.5% PEF group, respectively, with the lowest values observed in 7.5% PEF patties through the storage days. The lower L∗ and a∗ values are expected from the dark and purple color.

The TBARS is the most widely used indicator for lipid oxidation in meat and meat products. From Table 5, the raw PEF patties had significantly lower TBARS (*p* < 0.05) than the control group during retail display at 1, 3, 5, and 7 days, respectively. TBARS increased (*p* < 0.05) for all treatment groups with the increase in storage time. However, TBARS value rapidly increased in the control patties during days 3 to 5 of retail display. The maximum TBARS was 5.86 *μ*g/g in the raw control patties on day 7. The TBARS at days 1 to 7 of display storage in PEF beef patties were lower than those in beef patties without PEF. These results were associated with the presence of anthocyanin and phenolic compounds, which may improve the oxidative stability of beef patties over display storage. Previous studies reported that nasunin, phenolic compound and anthocyanin pigment of eggplant skin, acts as an antioxidant by scavenging lipid radicals, inhibition of hydroxyl radical generation, and inhibition of superoxide scavenging activity [42]. According to the color changes of the patties, the reduction in a∗ value during storage time was due to the change of oxymyoglobin to metmyoglobin, leading to increased brown coloring in meat product [43]. Moreover, less change of L∗ and a∗ value was also observed in PEF patties over the control patties. Consequently, the 7.5% PEF beef patties had a more consistent a∗ value during the storage while the a∗ value of control decreased rapidly, indicating that meat color was altered by antioxidant properties of PEF. Uthumporn et al. [6] also documented that the extract of purple eggplant peels showed high free radical scavenging capacity and prevented the propagation of lipid oxidation. No differences (*p* < 0.05) in b∗ values were found among the treatments although the values were increased with the increase of the PEF level in day 0 throughout the storage. In this study, the TBARS value of patty with 7.5% PEF maintained the level (1.89 *μ*g/g sample) lower than the acceptable limit (2 *μ*g/g sample) where the rancid overpowered beef flavor [44]. TBARS value over 1.5 *μ*g MDA/g sample might have a negative effect on consumer health [45].

### 3.5. Sensory Characteristics

The sensory scores of PEF beef patties are presented in Table 6. The sensory panel scores for beef patties were affected by amount of PEF. The beef patties with 5 and 7.5% PEF had significantly (*p* < 0.05) higher tenderness scores than the 0% PEF control. The juiciness scores of the PEF beef patties were lower than control (*p* < 0.05). This could be due to less fat in PEF beef patties resulted in drier mouthfeel after tasting. Similar results were reported by Selani et al. [46] that beef burgers with 10% passion fruit lowered tenderness and juiciness scores compared to control burgers. Fat has considerable importance to the texture and juiciness of meat products. The tenderness score decreases when fat is reduced in product formulation [2]. The addition of PEF seemed to reduce the changes in sensory properties caused by fat reduction. It was reported that dietary fibers reform the meat-protein matrix resulting in decreasing the gel strength [47]. The hardness varied inversely with water retention of meat product [48]. In this study, however, the combination of protein and PEF may improve tenderness and juiciness of the beef patties. In off-flavor evaluation, all PEF patties showed no objection except the level of 7.7% PEF probably due to the specific piquant and bitter taste of saponins and glycoalkaloids in eggplant [49]. The appearance score of PEF beef patties was comparatively lower (*p* < 0.05) than control beef patties, potentially due to the dark purple color of PEF. Likewise, dark and dry appearance products were often rejected by consumer and therefore resulted in a loss of revenue to the meat industry [50]. Nevertheless, no significant differences (*p* > 0.05) were found in odor, texture, and taste scores among all beef patties.

PEF addition seems to interfere with the overall acceptability score since there was significant difference between the PEF and control beef patties. The sample with the highest PEF level was less accepted (*p* < 0.05) than the other patties. This result agreed with the previous results of the patties containing hazelnut pellicle and oat flour [42, 44]. The increase in hardness was observed in the analysis of the instrumental texture profile (Table 4). Consumer acceptability of meat products decreased as fiber content increased especially over 3% by weight [51]. However, the results of our study showed that the addition of PEF up to 5% did not result in the decrease of the overall acceptability compared to the control patties. Thus, it will be ideal to add PEF to beef patties for 5% or less.

## 4. Conclusions

Our findings revealed that PEF has the potential to be used as an ingredient for fat replacer, antioxidant, and fiber enhancer. Increasing the PEF up to 7.5% on beef patties markedly reduced fat content and enhanced the dietary fiber content in meat products. The use of PEF increased the water- and oil-holding capacity, product yield, and cooking yield. Fat substitution with PEF up to 7.5% improved sensory attributes, especially juiciness score. However, the optimum content of PEF in beef patties was 5.0% in order to maintain the score of visual appearance, juiciness, hardness, and flavor. Based on those results, PEF can be considered as a promising functional ingredient in beef patties to improve product quality and shelf-life.

## Figures and Tables

**Table 1:** Formulation of beef patties containing PEF.

Treatments	Ingredients (%)							Notation
	PEF	Lean meat	Water	Beef fat	Salt	Pepper	Seasoning	
Control	0	70	5	20	1	1	3	Commercial formulation
1	2.5	70	5	17.5	1	1	3	12.5% reduced-fat formulation
2	5	70	5	15	1	1	3	25% reduced-fat formulation
3	7.5	70	5	12.5	1	1	3	37.5% reduced-fat formulation

**Table 2:** Nutritional compositions of PEF, raw, and cooked beef patties containing different concentrations of PEF (% wet basis).

Parameters	Moisture (%)	Protein (%)	Fat (%)	Ash (%)	Crude fiber (%)	Carbohydrate (%)^∗^	Total energy value (kcal)^∗∗^
Purple eggplant flour	5.21 ± 0.14	10.48 ± 0.42	1.87 ± 0.05	7.45 ± 0.26	12.64 ± 0.71	62.35 ± 0.27	322.72 ± 6.58
*Raw beef patties*							
Control	56.75 ± 0.22	18.91 ± 0.18*a*	23.35 ± 0.42^a^	1.00 ± 0.16^b^	ND	—	286.53 ± 5.24^a^
2.5% PEF	56.77 ± 0.52	17.56 ± 0.17^b^	20.89 ± 0.36^b^	1.29 ± 0.52^b^	0.31 ± 0.01^c^	3.18 ± 0.71^b^	271.12 ± 4.26^b^
5% PEF	58.57 ± 0.66	15.24 ± 0.20^c^	17.68 ± 0.26^c^	2.79 ± 0.30^a^	0.65 ± 0.24^b^	5.07 ± 0.29^b^	241.70 ± 7.41^c^
7.5% PEF	58.88 ± 0.11	14.49 ± 0.11^d^	16.18 ± 0.66^d^	3.02 ± 0.32^a^	0.91 ± 0.55^a^	6.52 ± 1.22^a^	228.66 ± 6.12^d^
*Cooked beef patties*							
Control	53.15 ± 1.65	22.19 ± 0.18^a^	22.77 ± 0.42^a^	1.89 ± 0.14^c^	ND	—	294.93 ± 4.25^a^
2.5% PEF	54.48 ± 0.88	21.22 ± 0.17^b^	17.18 ± 0.27^b^	2.29 ± 0.52^b^	0.52 ± 0.01^c^	4.31 ± 0.01^c^	256.64 ± 5.12^b^
5% PEF	55.15 ± 0.65	19.92 ± 0.20^c^	15.09 ± 0.09^c^	2.58 ± 0.30^a^	0.98 ± 0.16^b^	6.28 ± 0.12^b^	240.65 ± 6.25^c^
7.5% PEF	55.83 ± 0.73	17.69 ± 0.11^d^	14.53 ± 0.68^d^	3.42 ± 0.32^a^	1.22 ± 0.75^a^	7.31 ± 0.24^a^	231.57 ± 4.12^d^

Values are mean ± SD of 3 determinants, sample size (*n*) = 5. ^a,b,c,d^Means in the same column followed by different letters are significantly different (*p* < 0.05). PEF: purple eggplant flour; ND: not detected. ^∗^Carbohydrate (%) = 100 − (%moisture + %ash + %fat + %protein + %crude fiber). ^∗∗^Total energy value (kcal/100 g) = (%fat × 9) + (%protein × 4.02) + (%carbohydrate × 3.8).

**Table 3:** Cooking properties of cooked beef patties with different concentrations of PEF.

Parameters	Cooking yield (%)	Moisture retention (%)	Fat retention (%)	Diameter reduction (%)	Thickness reduction (%)
Control	74.16 ± 0.22^b^	38.41 ± 1.16^d^	82.31 ± 0.67^c^	20.02 ± 0.24^a^	3.59 ± 0.62^a^
2.5% PEF	84.20 ± 0.29^a^	45.03 ± 0.59^c^	86.00 ± 0.49^b^	16.22 ± 0.15^b^	2.94 ± 0.70^b^
5% PEF	84.64 ± 0.18^a^	47.70 ± 0.58^b^	87.03 ± 1.10^a^	15.74 ± 0.30^b^	2.84 ± 0.35^b^
7.5% PEF	85.44 ± 0.22^a^	50.43 ± 0.65^a^	87.59 ± 1.90^a^	11.33 ± 0.47^c^	2.81 ± 0.31^b^

Values are mean ± SD of 3 determinants, sample size (*n*) = 5. ^a,b,c,d^Means in the same column followed by different letters are significantly different (*p* < 0.05). PEF: purple eggplant flour.

**Table 4:** Instrumental textural properties of raw and cooked beef patties containing different concentrations of PEF.

Parameters	*Raw patties*	*Cooked patties*				
	Hardness (N)	Springiness	Chewiness N∗mm	Cohesiveness	Firmness (kg)	Shear force value (N)
Control	2.28 ± 0.70^b^	0.90 ± 0.03	201 ± 24^b^	0.21 ± 0.04^b^	0.30 ± 0.06	38.3 ± 0.36
2.5% PEF	2.67 ± 0.57^a^	0.86 ± 0.31	268 ± 45^b^	0.23 ± 0.03^b^	0.34 ± 0.05	36.8 ± 0.23
5% PEF	2.85 ± 0.53^a^	0.85 ± 0.15	315 ± 57^a^	0.24 ± 0.06^b^	0.33 ± 0.02	35.9 ± 0.06
7.5% PEF	2.97 ± 0.19^a^	0.91 ± 0.10	387 ± 68^a^	0.27 ± 0.01^a^	0.33 ± 0.01	35.1 ± 0.04

Values are mean ± SD of 3 determinants, sample size (*n*) = 5. ^a,b,c,d^Means in the same column followed by different letters are significantly different (*p* < 0.05). PEF: purple eggplant flour; SEM: standard error of the mean.

**Table 5:** Color stability and lipid oxidation of beef patties containing different concentrations of PEF during retail display storage.

Items	Storage time (day)	Treatment			
		Control	2.5% PEF	5% PEF	7.5% PEF
L∗	Day 0	41.87 ± 0.37^Ac^	40.39 ± 0.32^Bd^	38.70 ± 0.57^Cc^	37.91 ± 0.52^Cc^
	Day 1	41.69 ± 0.57^Ac^	40.96 ± 0.02^Ac^	39.13 ± 0.59^Bc^	38.63 ± 0.12^Bc^
	Day 3	42.72 ± 0.41^Ab^	42.96 ± 0.02^Ab^	40.91 ± 0.13^Bb^	39.83 ± 0.54^Cb^
	Day 5	43.07 ± 0.06^Ab^	43.43 ± 0.38^Aa^	41.74 ± 0.24^Ba^	40.64 ± 0.30^Ca^
	Day 7	45.07 ± 0.44^Aa^	43.69 ± 0.27^Ba^	42.28 ± 0.26^Ca^	41.19 ± 0.55^Da^

a∗	Day 0	15.70 ± 0.28^Aa^	12.69 ± 0.15^Ba^	10.32 ± 0.26^Ca^	8.28 ± 0.54^Da^
	Day 1	14.79 ± 0.65^Ab^	12.11 ± 0.29^Ba^	9.90 ± 0.13^Ca^	7.86 ± 0.13^Da^
	Day 3	12.56 ± 0.12^Ac^	9.67 ± 0.65^Bb^	8.98 ± 0.65^Bb^	7.16 ± 0.08^Cb^
	Day 5	10.32 ± 0.31^Ad^	8.50 ± 0.55^Bc^	8.40 ± 0.25^Bb^	6.98 ± 0.09^Cb^
	Day 7	8.91 ± 0.27^Ae^	8.07 ± 0.34^Bc^	7.32 ± 0.12^Cc^	6.68 ± 0.07^Db^

b∗	Day 0	5.94 ± 0.49^C^	7.84 ± 0.32^B^	8.92 ± 0.20^A^	9.08 ± 0.40^A^
	Day 1	6.25 ± 0.64^C^	7.55 ± 0.46^B^	8.19 ± 0.50^AB^	8.69 ± 0.43^A^
	Day 3	6.25 ± 0.56^B^	7.95 ± 0.55^A^	8.20 ± 0.09^A^	8.38 ± 0.41^A^
	Day 5	6.01 ± 0.54^C^	7.62 ± 0.41^B^	8.68 ± 0.36^A^	8.93 ± 0.75^A^
	Day 7	6.14 ± 0.18^B^	7.70 ± 0.25^A^	8.52 ± 0.06^A^	8.00 ± 0.77^A^

TBARS (*μ*g MDA/g)	Day 0	0.06 ± 0.02^d^	0.05 ± 0.01^d^	0.06 ± 0.01^d^	0.05 ± 0.02^d^
	Day 1	0.51 ± 0.06^Ad^	0.45 ± 0.08^Ad^	0.21 ± 0.10^Bd^	0.06 ± 0.03^Cd^
	Day 3	1.80 ± 0.15^Ac^	1.23 ± 0.28^Bc^	0.95 ± 0.12^Bc^	0.41 ± 0.16^Cc^
	Day 5	4.27 ± 0.44^Ab^	2.38 ± 0.24^Bb^	2.01 ± 0.04^Bb^	1.89 ± 0.05^Bb^
	Day 7	5.86 ± 0.46^Aa^	3.60 ± 0.42^Ba^	3.09 ± 0.59^BCa^	2.28 ± 0.09^Ca^

Values are mean ± SD of 3 determinants, sample size (*n*) = 5. ^a,b,c,d,e^Different letters indicate a significant difference between storage time (*p* < 0.05). ^A,B,C,D^Means in the same row followed by different letters are significantly different (*p* < 0.05). PEF: purple eggplant flour; TBARS: thiobarbituric acid-reactive substances.

**Table 6:** Sensory characteristics of beef patties containing different concentrations of PEF.

Parameters	Tenderness	Juiciness	Off-flavor	Appearance	Color	Odor	Texture	Taste	Overall acceptability
Control	6.04 ± 0.20^b^	7.18 ± 0.59^a^	1.34 ± 0.39^c^	7.01 ± 1.16^a^	7.20 ± 1.03^a^	6.93 ± 0.77	7.13 ± 0.52	7.07 ± 0.57	6.63 ± 0.43^a^
2.5% PEF	6.16 ± 0.94^ab^	6.94 ± 0.57^ab^	1.79 ± 0.65^b^	6.91 ± 0.72^a^	6.25 ± 0.91^b^	6.60 ± 0.90	6.23 ± 0.92	7.78 ± 0.55	6.46 ± 0.44^a^
5% PEF	6.21 ± 0.67^ab^	6.96 ± 0.30^ab^	1.93 ± 0.48^b^	6.33 ± 0.75^b^	6.04 ± 1.01^b^	6.23 ± 0.79	6.26 ± 0.65	7.14 ± 0.03	6.31 ± 0.39^a^
7.5% PEF	6.59 ± 0.47^a^	6.71 ± 0.64^b^	2.32 ± 0.58^a^	5.79 ± 0.78^b^	5.98 ± 0.93^b^	5.48 ± 0.76	6.33 ± 0.65	6.84 ± 0.44	6.07 ± 0.64^b^

Values are mean ± SD of 3 determinants, sample size (*n*) = 10. ^a,b,c,d^Means in the same column followed by different letters are significantly different (*p* < 0.05). ^1^Nine-point scale for tenderness, juiciness, and off-flavor (1 = extremely hard, dry, and no off-flavor; 9 = extremely soft, juicy, and extremely high off-flavor) and nine-point scale for appearance, color, odor, texture, taste, and overall acceptability (1 = extremely dislike, 9 = extremely like). PEF: purple eggplant flour.

## Data Availability

Data used to support the findings of this study are included within the article.
